# Falling and Landing Framework (FLF): A Consensus on a Novel Falling and Landing Video Analysis Framework for Use Across Rugby Codes

**DOI:** 10.1002/ejsc.70015

**Published:** 2025-07-12

**Authors:** Freja J. Petrie, James Stephen Woodward, Shreya McLeod, Stephen William West, Danielle Salmon, Andrew J. Gardner, Isla J. Shill, Janelle Romanchuk, Kathryn Dane, Matthew Kitchin, Ben Jones, Kelly A. Mackintosh, Chelsea Starbuck, Sharief Hendricks, Gemma Philips, Sam Jones, Gregory Tierney, Melitta A. McNarry

**Affiliations:** ^1^ Assistive Technology Innovation Centre (ATiC) University of Wales Trinity St David Swansea UK; ^2^ School of Sport Ulster University Belfast UK; ^3^ Carnegie Applied Rugby Research (CARR) Centre Carnegie School of Sport Leeds Beckett University Leeds UK; ^4^ Discipline of Physiotherapy School of Allied Health Australian Catholic University Brisbane Australia; ^5^ School of Medicine and Public Health University of Newcastle Newcastle Australia; ^6^ Sport Injury Prevention Research Centre Faculty of Kinesiology University of Calgary Calgary Canada; ^7^ World Rugby Injury Prevention and Player Welfare Dublin Ireland; ^8^ Sydney School of Health Sciences Faculty of Medicine and Health University of Sydney Camperdown Australia; ^9^ School of Physical Education, Sport, and Exercise Science University of Otago Dunedin New Zealand; ^10^ New Zealand Rugby Wellington New Zealand; ^11^ Discipline of Physiotherapy School of Medicine Trinity College Dublin Dublin Ireland; ^12^ England Performance Unit Rugby Football League Etihad Campus Manchester UK; ^13^ School of Behavioural and Health Sciences Faculty of Health Sciences Australian Catholic University Brisbane Australia; ^14^ Division of Physiological Sciences Department of Human Biology Faculty of Health Sciences University of Cape Town Cape Town South Africa; ^15^ Premiership Rugby London UK; ^16^ Applied Sports, Technology, Exercise and Medicine Research Centre Swansea University Swansea UK; ^17^ Hull Kingston Rovers Hull UK; ^18^ Uno‐X Mobility Pro Cycling Oslo Norway; ^19^ School of Engineering Ulster University Belfast UK

**Keywords:** analysis, game analysis, injury and prevention, team sport

## Abstract

Understanding how players experience head‐acceleration events (HAE) whilst playing rugby is a priority area of research. In both rugby union and league, video analysis frameworks have been developed to comprehensively define key features of contact events. However, these frameworks were developed prior to recent advances in our understanding regarding the proportion of HAEs that occur due to head‐to‐ground mechanisms and do not consider important post‐contact variables. Therefore, there is a need to supplement the existing frameworks in order to capture how players fall and land post‐tackle. This study used the Delphi method with an interdisciplinary, international team of researchers, coaches and video analysts (working with a variety of playing levels in rugby union and league) to establish a consensus for defining falling and landing events. Subsequently, a draft framework was developed on which the research team provided feedback via online meetings, culminating in the falling/landing framework that each member of the research team rated agreement on, via a nine‐point Likert‐type scale, with consensus deemed to be reached when the median score was ≥ 7. The median scores were 8.0 (7.8–8.0), 8.0 (7.0–9.0) and 8.0 (8.0–9.0) for ‘Additional Contextual Characteristics for Carry and Tackle Events,’ ‘Falling Characteristics of Tackle and Carry Events,’ and ‘Landing Characteristics of Tackle and Carry Events,’ respectively. This novel framework defines more comprehensive falling and landing variables to capture post‐contact injury and performance markers in both rugby union and league, through a standardised approach.

## Summary

- This consensus study advances previous frameworks and enables vital falling and landing related aspects of head acceleration events to be captured in a standardised manner.
- These variables should be used alongside existing rugby video analysis frameworks (e.g., RUVAC, video analysis framework for the rugby league tackle) to identify potential injury and performance markers throughout all phases of the tackle.
- Recommendations are provided for contextualising video‐analysis findings with complimentary sociodemographic, technological and performance measures.

## 1 Introduction

Video analysis has been extensively used in rugby, with its practicality and financial viability enabling it to inform injury prevention strategies and performance analyses (Tucker et al. 2017; S. W. West et al. 2022; S. West et al. 2021; Shill et al. 2023; den Hollander et al. 2018). To ensure consistency between studies, video‐analysis consensus frameworks are useful in defining and standardising key variables. In rugby specifically, the Rugby Union Video Analysis Consensus group (RUVAC) framework (Hendricks et al. 2020), and the video analysis framework for the rugby league tackle (Hopkinson et al. 2022), established a consensus for pre‐contact, contact and, to a lesser extent, post‐contact injury and performance variables. Although comprehensive, these frameworks were published prior to the studies that reported the propensity for head‐acceleration events (HAEs) to occur in the falling/landing phase of the tackle/carry (Woodward et al. 2022; Tooby et al. 2023; Williams et al. 2022). Subsequently, the post‐contact events in the aforementioned frameworks are limited to ‘grounding of the ball carrier,’ ‘orientation of ball carrier at initial landing,’ and ‘body region ball carrier landed on’ in rugby league (Hopkinson et al. 2022), and ‘tackler leg drive after contact,’ ‘upper body usage after contact,’ and ‘jackal’ in rugby union (Hendricks et al. 2020). However, given that recent research has highlighted the significance of head‐to‐ground and landing‐induced whiplash‐style HAE and head injury mechanisms, these post contact variables would not capture potential risk factors (Williams et al. 2022).

Sports such as gymnastics and American Football have investigated mechanisms of fall‐related injuries. These studies have focused on the measurement of head‐to‐ground HAEs rather than establishing variables to describe the falling and landing stages (Bagherian et al. 2025; Kent et al. 2020; Pritchard et al. 2020). In wrestling and judo, falls are recognised as a mechanism of injury, but the variables within these falls are not described in depth (Shadgan et al. 2024; Vasilescu et al. 2023; Arkkukangas et al. 2020; Sakuyama et al. 2021; Jadczak et al. 2024). Video analysis frameworks capturing specific falling and landing variables have been developed in equestrian sports; however, their highly specialised nature limits applicability into rugby union and league (Nylund et al. 2021, 2022).

Falling and landing can be a key tactical element of the sport, given that in both rugby league and rugby union, a phase of play will typically be terminated by a fall to the ground (World Rugby 2023; Rugby Football League 2021). Despite falling/landing happening often in both rugby codes, the relevance of this contact stage to injury has not been acknowledged in video analysis literature until recently (Woodward et al. 2022; Tooby et al. 2023; Williams et al. 2022). In the British university rugby union, 26.1% of female and 9.7% of male HAEs were caused by head‐to‐ground contact post‐fall (Williams et al. 2022). Within these head‐to‐ground HAEs, 78.0% of the female events and 0.5% of the male events were associated with whiplash‐style head kinematics (Williams et al. 2022). Similarly, in a Canadian university female rugby union cohort, head‐to‐ground contact was the cause of 35.0% of concussive HAEs (S. W. West et al. 2022). In the English Women's Super League (rugby league), head‐to‐ground contact had a propensity of 26.1 (17.1–38.2) per 1000 tackle events (Spiegelhalter et al. 2023). Across the first three seasons of the National Rugby League Women's Premiership in Australia, 33.0% of head‐impact events were experienced by a falling or diving ball carrier (McLeod et al. 2023). In addition to injury occurrence and prevention, a more comprehensive understanding of falling mechanisms is also important from a tactical perspective, particularly in rugby union where, unlike rugby league, possession of the ball can be contested at the ruck (World Rugby 2023; Rugby Football League 2021). Qualitatively, the falling and landing stage of the tackle has been identified as a knowledge gap by rugby coaches, and the importance of learning to fall safely and effectively has been highlighted by players (Stodter et al. 2023; Dane et al. 2024). It is therefore crucial that video‐analysis frameworks identify falling and landing variables to further explore injury mechanics and performance outcomes. Thus, the aim of this study was to engage a wide range of rugby researchers, players, and support staff to establish a consensus framework for falling and landing variables that can be used standalone and for supplementing pre‐existing frameworks, such as the RUVAC (Hendricks et al. 2020) and video analysis framework for the rugby league tackle (Hopkinson et al. 2022).

## 2 Methods

### 2.1 Panel Selection

To establish a consensus on the key variables for describing the falling/landing stage of tackle and carry events, 23 people professionally involved in rugby (players, coaches, referees, physiotherapists and medical staff, researchers, and video analysts) were recruited through established email networks. Of the 23 contacted, 18 responded to the first round of the consensus procedure and 15 responded to the final round. Characteristics of the respondents can be found in Table 1. No formal evidence review was undertaken, given this consensus piece builds upon the previous synthesis of rugby union and rugby league video‐analysis frameworks (Hendricks et al. 2020; Hopkinson et al. 2022). Ethics approval was granted by Ulster Sports and Exercise Science Research Institute Ethics Committee (project number: SESRI‐23‐011‐A).

**TABLE 1:** Research team characteristics.

Characteristics	Representation in research team
Genders of research team	Women (*n* = 10, 60.0%) Men (*n* = 7, 33.3%) Non‐binary (*n* = 1, 6.7%)
Genders of the research team's study populations	Women (*n* = 5, 40.0%) Men (*n* = 0, 0.0%) Women and men (*n* = 13, 60.0%)
Nationalities of the research team's study populations	United Kingdom (*n* = 10, 55.6%) Irish (*n* = 1, 5.6%) Australian (*n* = 2, 11.1%) Canadian (*n* = 1, 5.6%) New Zealand (*n* = 1, 5.6%) International (*n* = 3, 16.7%)
Rugby codes researched^a^	Union (XV) (*n* = 13, 59.1%) League (*n* = 4, 18.2%) Rugby 7s (*n* = 1, 4.5%) Union and league (*n* = 4, 18.2%)
Research team's additional roles within rugby^a^	Coaching (*n* = 5, 19.2%) Refereeing (*n* = 1, 3.8%) Playing (*n* = 4, 15.4%) Physio and medical (*n* = 5, 19.2%) Video analyst (*n* = 2, 7.7%) No additional role (*n* = 9, 34.6%)
Playing level of research team's study populations^a^	University (*n* = 5, 18.5%) Amateur (*n* = 9, 33.3%) Elite (*n* = 7, 25.9%) Youth (*n* = 2, 7.4%) All (*n* = 4, 14.8%)

^a^ Some researchers are involved across multiple rugby codes, roles and playing levels.

### 2.2 Consensus Process

Consistent with the framework developed by Hendricks et al. (Hendricks et al. 2020), a Delphi consensus method was used to establish agreement on a novel framework (McMillan et al. 2016). The Delphi method is primarily used as it facilitates and supports structured collaboration amongst experts from a wide range of disciplines (Jones and Hunter 1995). Initial meetings were held with a core group of the research team (J.W., F.P., I.S., S.W., D.S., J.R.) to discuss ideas for variables related to falling and landing in rugby union and create a first working draft. Following this, a meeting was held with a group of researchers in rugby league (J.W., F.P., S.M., M.K.) to ensure that the initial draft framework could be applicable across rugby codes. Once these variables were checked for coherency between rugby league and union, an online survey was implemented and sent to additional researchers to evaluate initial consensus (LimeSurvey GmbH, Hamburg, Germany). This survey defined all the variables in the draft framework, subsequently termed the falling/landing framework (FLF). All members of the research team were asked to rate the ‘extent to which the inclusion of this category and its definitions would be valuable in the framework’ via a nine‐point Likert‐type scale. Nine‐point scales are ‘often’ used for their greater criterion validity, sensitivity, and participant preference compared to scales with fewer levels (McMillan et al. 2016; Taherdoost 2019). As proposed by Fitch et al. (Fitch et al. 2001), the pre‐determined threshold required for group consensus was a median Likert score greater than, or equal to, seven. In addition to this Likert‐type scale, further feedback could be provided by participants via a free‐text box. Once the research team had completed the survey, median agreement ratings and interquartile ranges (IQRs) were calculated in MATLAB (MATLAB_R2024b; MathWorks, Massachusetts, USA).

Whilst a consensus was reached following the first survey, the research team reported opportunities to refine the framework via the free‐text boxes. The suggestions provided in the free‐text boxes related to study practicalities (*n* = 9, 24%), variable terminology (*n* = 3, 8%) and variable definitions (*n* = 25, 68%). To explore the suggestions made, a series of online meetings were conducted in November 2023 (via Zoom Video Communications Inc., California, USA) to discuss and refine framework definitions. These meetings were attended by 13 research members, recorded and made available to all group members to aid transparency. If members were unable to attend or complete the survey, they provided comprehensive feedback via email. The feedback was amalgamated, and the initial survey updated to reflect the proposed changes to the framework. The updated survey was re‐distributed and completed by the wider research team. Median agreement ratings and IQRs were calculated (as above) and final consensus was reached.

### 2.3 Reliability

In line with Hopkinson et al.’s (Hopkinson et al. 2022) approach, reliability of the framework was examined by conducting an inter‐rater and intra‐rater reliability test on a randomly selected sample of 30 tackles, from publicly available footage from one match of the 2023/24 playing season of the British Universities and Colleges Sport (BUCS) National League. Intra‐rater reliability was conducted on the same sample of 30 tackles, with a repeated analysis 7 days later, by JW (3 years of rugby coding experience) using Kinovea (v.0.9.5) (Hopkinson et al. 2022; Wheeler et al. 2010). For inter‐rater reliability, FP (3 years of rugby coding experience) coded the same sample of tackles. To examine reliability across rugby codes, a subsequent inter‐rater reliability test was conducted on a randomly selected sample of 30 tackles, from publicly available footage from one match of the 2024/25 playing season of the men's National Rugby League. Kappa (*κ*) values were calculated in MATLAB (MATLAB_R2024b; MathWorks, Massachusetts, USA) and used to determine the reliability of each analysis variable for each tackle in the sample (James et al. 2007), where a *κ* value of 0.60–0.79 represented *moderate agreement*, 0.80–0.89 *strong agreement* and 0.90–0.99 *near perfect agreement* (O’Donoghue 2014). The intra‐ and inter‐rater agreement scores for each analysis variable can be found in Table 2.

**TABLE 2:** Description of variables, and reliability analysis for the consensus framework for rugby union (RU) and rugby league (RL).

Additional contextual characteristics for carry and tackle events					
Variables	Descriptors	Delphi consensus score (median (IQR))	Women's BUCS rugby union‐ intra‐rater (*κ*)	Women's BUCS (RU) inter‐rater (*κ*)	Men's super league (RL) ‐ inter‐rater (*κ*)
Fend strategy	Forearm—Carrier fends tackler with forearm. Hand—Carrier fends tackler with open palm. Fist—Carrier fends tackler with a closed fist. Bump—Carrier bumps tackler with tucked ball/forearm. None—No fend strategy employed.	8.0 (7.5–8.25)	0.82	0.87	0.79
Offload strategy	One hand—The carrier offloads the ball with one hand. Two hand—The carrier offloads the ball with both hands. No offload—The carrier takes the ball to the floor. Drop—Ball is dropped.	8.0 (7.75–8.0)	0.90	0.90	1.00
Falling characteristics of tackle and carry events					
Direction of fall	Forwards—The player falls forwards. Backwards—The player falls backwards. Sideways—The player falls sideways. Drop—The player drops down onto their knees or rear. No fall—The player doesn't fall or go to the ground.	8.0 (8.0–9.0)	0.85	0.71	0.87
Trajectory to ground	Interrupted—The player's fall to the ground is interrupted by a player/object. Uninterrupted—The player's fall to the ground is uninterrupted.	8.0 (7.0–9.0)	0.80	0.71	0.72
Neck position maintenance (e.g. whiplash)	Controlled—Neck position is controlled during the fall/landing. Uncontrolled/Whiplash—Neck position is not controlled during the fall/landing (whiplash).	8.0 (7.5–9.0)	0.86	0.75	0.76
Location of hands during fall	None free—Both of the player's hands are obstructed or wrapped up by the tackler. One free—One of the player's hands is free to move. Both free—Both of the player's hands are free to move.	8.0 (7.3–8.8)	0.73	0.81	0.80
Landing characteristics of tackle and carry events					
Landing strategies	Controlled—The player attempts to control their landing. Visible actions may include: Catching oneself with their upper limb (inclusive of hand, forearm, elbow or upper arm). Altering head or body position. Player‐initiated roll upon landing. Player twists body to avoid a backwards fall. Impeded—The player is impeded from making an attempt to control their landing by another player. This may occur when: The ball carrier is pushed to the floor by another player during or before the fall. The momentum of the tackle prevents any attempt to control the fall. Collision with another player on the ground disrupts attempts to control the fall. A smother tackle (Hendricks et al. 2020) is performed impeding protective use of the upper limbs. Uncontrolled—The player makes no attempt to control their landing. None of the variables listed in the impeded or controlled landing strategies are observed.	8.0 (7.5–9.0)	0.81	0.79	0.89
Landing position	Prone—The player lands face down. Supine—The player lands face up. Side—The player lands on their side. On rear—The player lands in a seated position. Knees—The player lands in a kneeling position. Rolling—The player rolls upon landing (if ball carrier, before presenting the ball at the ruck).	8.0 (8.0–8.3)	0.91	0.77	0.93
Location of hands at landing	None free—Both of the player's hands are obstructed. One free—One of the player's hands is free to move. Both free—Both of the player's hands are free to move.	8.0 (8.0–8.5)	0.85	0.85	0.76

## 3 Results

The final variables that achieved consensus (*n* = 9) were grouped into three main categories: ‘Additional Contextual Characteristics for Carry and Tackle Events’, ‘Falling Characteristics of Tackle and Carry Events’ and ‘Landing Characteristics of Tackle and Carry Events’. These variables, their consensus score, intra‐rater and inter‐rater scores are shown in Table 2.

**FIGURE 1:**
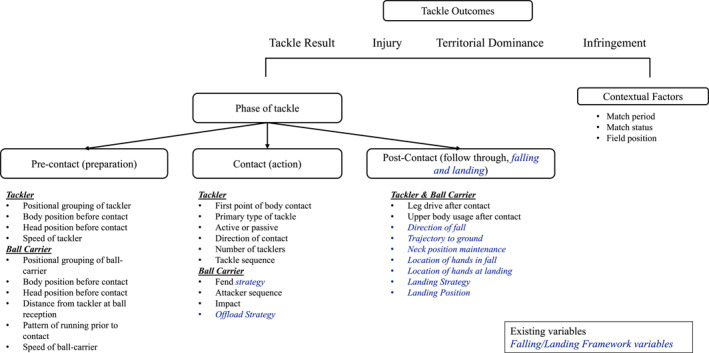
Adapted video analysis model for the tackle from Hendricks et al. (2020) with additional contextual and falling/landing framework (FLF) variables in blue italic font.

**FIGURE 2:**
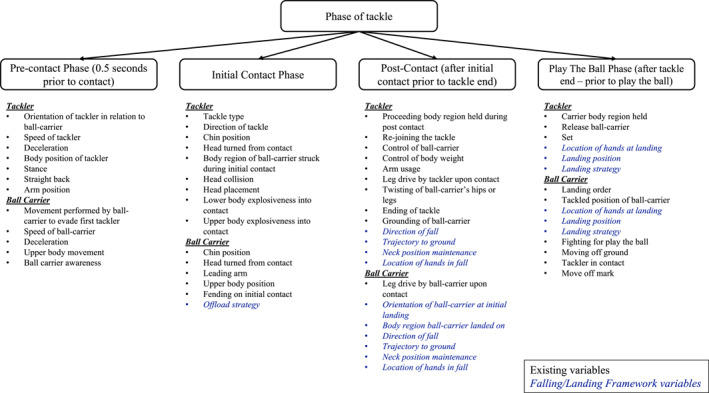
Adapted video analysis model for the tackle from Hopkinson et al. (2022) with additional and falling/landing framework (FLF) variables in blue italic font.

The incorporation of the FLF variables into the RUVAC and the Video Analysis Framework for the Rugby League Tackle is illustrated in Figures 1 and 2, respectively.

### 3.1 Areas of Disagreement

Not all variables achieved consensus. Specifically, ‘Time between the first frame of initial contact and the ball carrier coming to rest’, ‘Falling Sequence: List the three body parts that are the first to contact the floor’, ‘Fall body position’ and ‘Degree of neck flexion during fall’ achieved median scores and IQRs of 5.50 (3.0–7.0), 6.0 (4.0–7.0), 4.5 (3.0–6.0) and 6.0 (3.0–7.0), respectively. Following discussions amongst the authorship and during reliability testing, it became clear that whilst additional variables would make the framework more comprehensive, the inclusion of these variables would currently be impractical in the applied setting. As improved video technology is becoming increasingly available, these variables may be reconsidered in later iterations of frameworks.

## 4 Discussion

This study identified additional key falling/landing variables supplementary to the current consensus tackle‐analysis frameworks in rugby union and rugby league (Hendricks et al. 2020; Hopkinson et al. 2022) through the engagement of diverse and multi‐disciplinary rugby stakeholders. The diversity of the research team was of benefit to the quality of this framework. Insights were gathered from youth, university, community and elite rugby, from multiple nationalities. The incorporation of the FLF into current frameworks (e.g., RUVAC‐FL) will improve the inter‐study comparison between future rugby studies and provide further insights to performance and injury‐related variables in the post‐contact tackle phase. The final framework has a total of eight variables, with four from the initial proposed framework being removed due to a lack of consensus. This framework is a non‐exhaustive list that should be updated and added to in‐line with emerging evidence.

### 4.1 Research Implications

As with the RUVAC and Rugby League frameworks, variables from the FLF should be flexibly selected for inclusion in video analysis, guided by the specific research question of an individual study (Hendricks et al. 2020; Hopkinson et al. 2022). Recommendations for the type and quality of video used for analysis are discussed in‐depth by the authors of the RUVAC framework and detailed further by Shill et al. (Shill et al. 2023). Briefly, a single, roaming, zoom‐enabled camera positioned at a high vantage point is sufficient for analysis, particularly in amateur playing levels where the recording of multi‐angle broadcast‐quality footage is typically unfeasible (S. W. West et al. 2022). It should be acknowledged that as the variables of the FLF predominantly relate to activity nearer to the ground, inevitably, the density of players during formation of the ruck in rugby union may occlude a player from the field of view. Arguably, this occlusion would be reduced if multi‐angle footage could be analysed, however, pragmatic efforts should be made to represent all levels of rugby globally in video analysis literature, to guide the development of injury prevention strategies that are more representative of the wider playing population. In addition, to further investigate injury risk at all stages of the tackle, the FLF could also be incorporated within existing frameworks as standard.

Inter‐rater and intra‐rater reliability analysis highlighted a range of *moderate*, *strong* and near *perfect agreement* for the identified FLF variables. Despite the differences in playing contexts between Women's BUCS Rugby Union and Elite Men's Rugby League, inter‐rater reliability consistently achieved *moderate* and *strong agreement*, highlighting the applicability of the framework for use across playing levels and rugby codes. Although these reliability scores are relatively high, these may differ based on the coder's experience. Those who are planning on using the analysis framework should also conduct their own inter‐ and intra‐rater reliability. The meetings held with the research team also highlighted the benefit of combining the video analysis with additional data, such as GPS and instrumented mouthguards, which could also be used to enhance validity of analysed variables (Shill et al. 2023; Hendricks et al. 2020).

Falling/landing technique is a knowledge gap previously identified by coaches in rugby codes (Dane et al. 2024). Closing this knowledge gap remains challenging without sufficient research to inform practice. Therefore, this framework should be used to explore the mechanisms of the fall/landing relating to injury and performance outcomes. Previously, tackle proficiency scores have been established to investigate injury and performance outcomes in rugby union (Hendricks et al. 2018; den Hollander et al. 2019; Hollander et al. 2021). These studies have highlighted that highly proficient tackles are also tackles that were less associated with injury (Hendricks et al. 2018; Hollander et al. 2021). Using the proposed falling/landing framework (FLF), tackle proficiency scores could be extended to tackler and carrier post‐contact phases of the tackle. This would allow for a more comprehensive understanding of the proficiency and safety of the tackle event across all contact phases. It is important to note that where interventions are developed for improving tackle proficiency and safety, they should be informed by data that represents the wider playing population. For example, interventions designed and developed on elite‐level player data may not be applicable to amateur‐level players. In addition, interventions developed using data from men's rugby may be less applicable to women's rugby. This may be due to wider contextual factors such as sociocultural context, training and playing age, and tackle proficiency. For example, few studies acknowledge sociological factors such as the gendered environmental background, which has become an increasingly acknowledged pervasive factor in sports injury risk (Dane et al. 2024; Parsons et al. 2021; Dane et al. 2023; Petrie et al. 2024). Although there are proposed differences in HAE mechanisms in men's and women's rugby, a strength of this framework is that it offers standardised definitions of falling and landing variables that are designed for use in all contexts (Woodward et al. 2022; Tooby et al. 2023; Williams et al. 2022).

### 4.2 Limitations

The research group focus their work on countries that are ranked within the top 10 of men's and women's World Rugby rankings (World Rugby 2024). Language barriers precluded collaboration with non‐English speaking researchers. Efforts were made to engage with non‐rugby falling/landing experts during the design of the framework; however, none were available for collaboration at this time. Instead, literature was drawn from non‐rugby falling/landing research to support framework development. Further, a degree of selection bias was unavoidably present within this work, as it is likely that further recruitment of populations not represented in this consensus would enrich the framework and different outcomes may have been reached. Therefore, this framework should be considered a foundation that can be built upon as the inclusion of additional variables is justified by emerging research.

### 4.3 Conclusion

This new framework (FLF) has been developed via Delphi consensus and standardises variables pertaining to falling and landing in rugby codes. A diverse range of professionals was recruited who are involved in rugby union and league, in research, medical and performance roles. The FLF can be used concurrently with existing rugby video analysis frameworks to capture injury and performance outcomes across all contact phases. Use of these standardised frameworks, with the addition of the FLF‐identified falling/landing variables in rugby, will aid inter‐study comparison and support the development of future injury prevention and performance‐focused interventions.

## Author Contributions

F.P. and J.W. are Co‐PIs and joint first authors for this framework. J.W. is the guarantor. F.P., J.W., S.W., I.S., D.S., J.R. drafted the original draft framework. S.M., M.K., F.P. and J.W. advised on the suitability of draft variables for rugby league. All authors contributed to the preparation of this manuscript for publication.

## Equity, Diversity, and Inclusion Statement

The international author group is comprised from different disciplines within rugby codes (early career, senior researchers, medical staff, coaches, video analysts, and match officials). Ten authors identify as women, seven authors identify as men, and one identifies as non‐binary. Not all rugby playing nations were represented in the author group, which may influence the generalisability of the results to wider playing populations.

## Ethics Statement

Ethics approval was granted by Ulster Sports and Exercise Science Research Institute Ethics Committee (project number: SESRI‐23‐011‐A).

## Conflicts of Interest

J.W., G.T., S.W., I.S., A.G.,and S.H. and B.J. have received funding from World Rugby. G.P. is currently employed by the Rugby Football League, UK. S.H. is a consultant for World Rugby. A.G. is a contracted consultant for Rugby Australia. B.J. has also secured research funding from Rugby Football Union, Scottish Rugby, Premiership Rugby, Rugby Football League, Catapult Sports, Prevent Biometrics, HitIQ, Leeds Rhinos Rugby League, Yorkshire Carnegie Rugby Union, Bath Rugby, Wasps Rugby. B.J. is employed in a consultancy capacity by the Rugby Football League, and Premiership Rugby as their research lead. D.S. is employed by W.R. J.R. is employed by New Zealand Rugby. S.M. has no competing interests.

## Data Availability

Data is available upon reasonable request.
